# Pulp blood flow changes in maxillary and mandibular anterior teeth after orthodontic retraction: a prospective study

**DOI:** 10.1186/s12903-022-02559-7

**Published:** 2022-11-17

**Authors:** Runzhi Guo, Qianyao Yu, Yifan Lin, Jing Li, Yiping Huang, Weiran Li

**Affiliations:** 1 Department of Orthodontics, Peking University School and Hospital of Stomatology, 22 Zhongguancun Avenue South, Haidian District, 100081 Beijing, P.R. China; 2 Division of Paediatric Dentistry and Orthodontics, Faculty of Dentistry, the University of Hong Kong, Hong Kong SAR, China

**Keywords:** Bimaxillary protrusion, Tooth extraction, Laser-Doppler Flowmetry, Pupal blood flow

## Abstract

**Background:**

Previous studies of pulpal blood flow (PBF) changes in anterior teeth have been limited in the early phase of orthodontic treatment; less is known about the blood supply of anterior teeth in bimaxillary protrusion patients after orthodontic retraction.

**Methods:**

Fifty bimaxillary protrusion patients (25 orthodontic patients ready for debonding and 25 non-orthodontic patients) were selected as study participants. The PBF of maxillary and mandibular anterior teeth were measured using laser Doppler flowmetry. For orthodontic patients, the PBF was measured at 1 day (T1), 1 month (T2), and 3 months (T3) after fixed appliance removal. Non-orthodontic patient PBF was measured as a control. Cone-beam computed tomography (CBCT) examinations before and after orthodontic treatment were performed for orthodontic patients to measure the root resorption. The anterior teeth in orthodontic group were further divided into subgroups according to root resorption and patient age.

**Results:**

At T1 and T3, PBF changes did not differ significantly between the orthodontic and non-orthodontic groups. Maxillary lateral incisor, maxillary central incisor, and mandibular lateral incisor PBFs at T2 were significantly higher in the orthodontic group (*P* = 0.048, *P* = 0.04, and *P* = 0.021). No significant difference in PBF was found between the root resorption and non-resorption subgroups at any time point. Adolescent patients showed a higher PBF in the maxillary lateral incisor at T2 (12.23 ± 3.48) relative to that at T1 (9.10 ± 3.76) and T3 (9.81 ± 2.80) with statistically significant difference (*P* = 0.020).

**Conclusion:**

For bimaxillary protrusion patients with four premolars extraction, PBF in the maxillary anterior teeth increased transiently after orthodontic appliance removal and then returned to non-orthodontic levels 3 months later. This effect was more pronounced in adolescents. The PBF of anterior teeth after orthodontic retraction may not be influenced by root resorption.

## Background

Bimaxillary protrusion is characterized by proclined anterior teeth and protruding lips, which is common in patients with class I and class II malocclusion, especially in Asian population [1, 2]. To reduce the convexity of the profile and improve the lip competency in such cases, treatment plans often include the extraction of four premolars and retraction of the anterior teeth. The dental pulp status of anterior teeth after retraction treatment is an essential concern for orthodontists and endodontists. Histological studies have shown that orthodontic force can cause alterations in the dental pulp, such as circulatory disturbance, edema, vacuolization, and the induction of tissue fibrosis [3–5]. The dental pulp tissue may undergo processes ranging from circulatory vascular stasis to necrosis after orthodontic force application [6]. The prevalence of pulp necrosis after orthodontic treatment has been observed to range from 1 to 14% [7, 8]. While, some studies indicated that orthodontic force and tooth movement cause reversible metabolic changes in the dental pulp and do not induce the loss of dental pulp vitality [9–11]. At present, the pulp response to orthodontic treatment remains unclear.

Considering these influences of orthodontic force, the dental pulp vitality should be evaluated during and after orthodontic treatment. Commonly used pulp tests, such as electrical and thermal pulp tests, have limited ability to accurately reflect the pulp status [12]. These tests reflect only the pulpal neural response, which is subjective and unreliable. Laser Doppler flowmetry (LDF) is one of the most accurate and non-invasive methods for the verification of dental pulp vitality, as it involves the direct measurement of the PBF, rather than reliance solely on the patient response, as in other tests [12–14]. The principle of LDF is Doppler shift, which allows light beam to pass through the enamel and dentin toward the dental pulp and be reflected by the moving erythrocytes. Typically, the decrease in PBF is associated with a reduction in oxygen tension, which increases the possibility of pulp tissue injury [15, 16]. For bimaxillary protrusion patients, the blood supply to the dental pulp through the apical foramen could become compromised by long-distance retraction movement and long-term orthodontic force application [8, 17].

Currently, studies of PBF changes with anterior teeth movement have been mainly focused on the assessment of intrusive and extrusive movement effect [18–20]. Brodin et al. analyzed the effects of intrusion and extrusion forces on the PBF in the maxillary incisors using LDF, and found a temporary decrease in PBF after orthodontic intrusion [19]. Sabuncuoglu et al. also found that the change in PBF during the intrusion of the maxillary incisor with mini-implants was reversible, which decreased at 3 days and then increased at 3 weeks [20]. While, less is known about the blood supply of anterior teeth after long-distance retraction. Only one study has involved the examination of PBF changes in maxillary canines during retraction [21]; it revealed an increase in PBF during retraction, in contrast to other findings. To our knowledge, the PBF changes of anterior teeth in bimaxillary protrusion patients after retraction treatment have not been investigated.

Besides, most studies have focused on the effect of orthodontic force on the PBF in the early phase of orthodontic treatment (4 min–6 months after orthodontic force application) [11, 20, 22, 23]. Their results showed that orthodontic force was associated with a reduction in PBF, followed by a return to the pre-treatment level. Notably, the duration of orthodontic treatment is approximately 2 years, and long-term force application might jeopardize the pulp vitality of anterior teeth. Hence, the pulp status of anterior teeth undergoing long-distance retraction movements for extended periods of time should be investigated. The aim of this study is to examine PBF changes in maxillary and mandibular anterior teeth using LDF after orthodontic retraction, and to explore the effects of root resorption and age on PBF changes.

## Methods

### Participants

This prospective study was approved by the Peking University School and Hospital of Stomatology Ethics Committee (PKUSSIRB-202,168,141). All study participants or their guardians have provided written informed consent prior to enrollment. The bimaxillary protrusion patients who had completed orthodontic treatment and were ready for debonding, and non-orthodontic bimaxillary protrusion patients, were recruited in this study. The inclusion criteria were: (1) age 12–30 years, (2) bimaxillary protrusion (class I molar relationship, interincisal angle < 125°, lower and upper lips in advance of the E-line), (3) mild crowding (< 3 mm), (4) healthy periodontal and pulp statuses of the upper and lower anterior teeth before orthodontic treatment, and (5) complete root formation of the anterior teeth. The exclusion criteria were: (1) missing anterior teeth, (2) anterior-tooth crown or implant, (3) systemic disease, and (4) smoking. The orthodontic patients were treated by the same experienced orthodontist using pre-adjusted MBT appliances (3 M Unitek, Monrovia, CA, USA). The treatment involved the extraction of the four first premolars and retraction of the anterior teeth. The sliding mechanics with 0.019 × 0.025 inch stainless steel archwires were used for en masse retraction of anterior teeth. The bilateral retraction force was 150 g [24–26], and was reactivated every 4 weeks. Cone-beam computed tomography (CBCT) examinations before and after orthodontic treatment were performed to examine the root resorption. Non-orthodontic control patients were selected by age and sex matching.

### Sample size calculation

Sample size was calculated using Power Analysis and Sample Size for Windows software (PASS 2000, NCSS, Kaysville, UT, USA). Based on a previous study [22], a minimum sample size of 22 patients in each group were sufficient to detect the clinically significant differences (a PBF change of 0.98) with 80% power at 5% significance level (two-sided). To allow for possible dropouts during the study, a total of 50 bimaxillary protrusion patients, including 25 orthodontic patients and 25 non-orthodontic patients, were included in this study.

### Groups

**Fig. 1:**
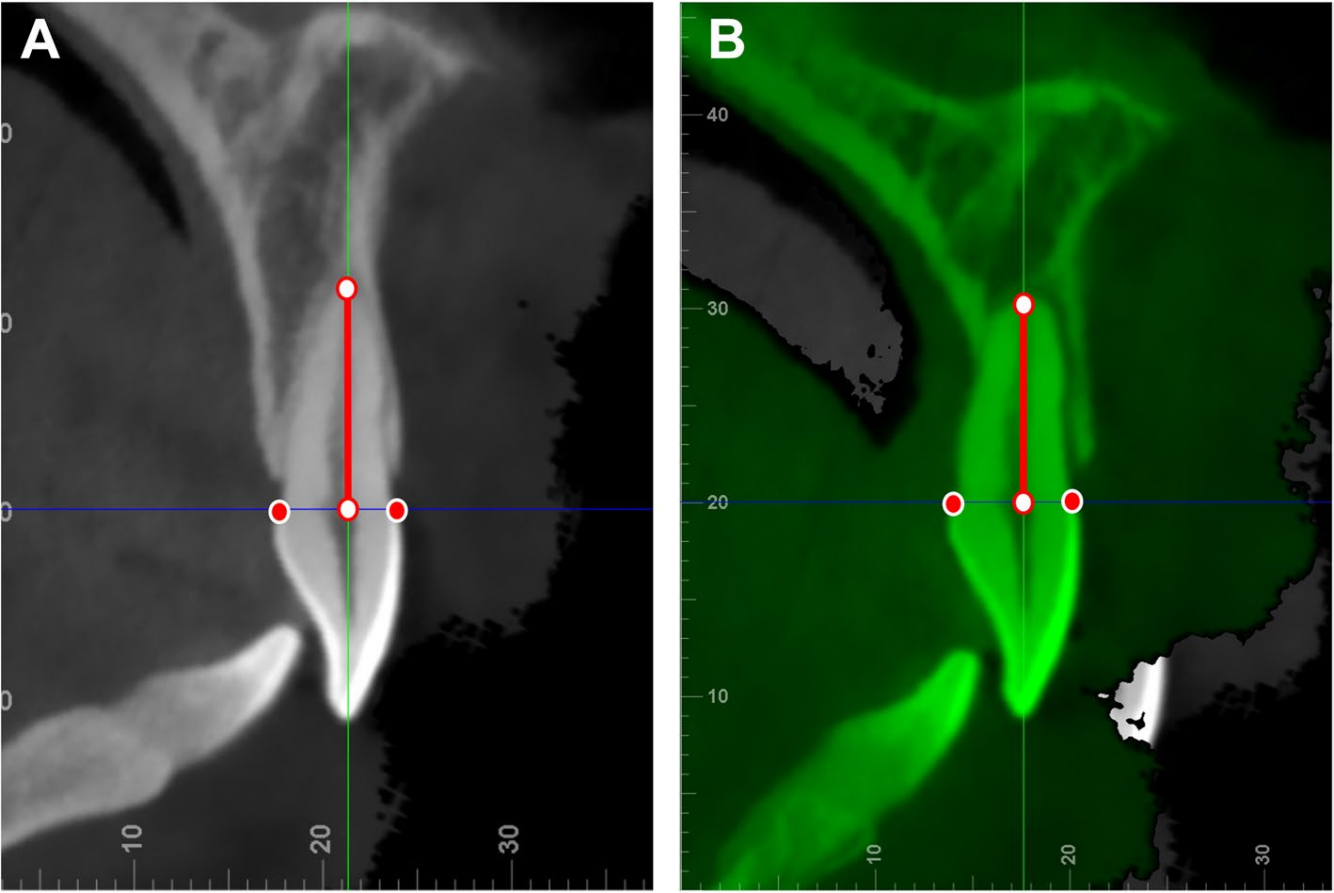
Diagram of root length measurement: vertical distance from the root apex to cementoenamel junction level (red line). **A**, pre-treatment root measurement; **B**, post-treatment root measurement

The PBF in maxillary and mandibular anterior teeth between Orthodontic group (OG) and Non-orthodontic group (NG) were firstly compared. To explore the effect of root resorption on PBF, the root lengths (distances between the cementoenamel junction and root apex) of orthodontic patients’ maxillary and mandibular anterior teeth were measured on pre- and post-treatment CBCT images (Fig. 1). Based on root resorption condition, the anterior teeth in orthodontic patients were then divided into Subgroup 1 (root resorption: root length decrease ≥ 1 mm, with the root appeared blunted with rounded apices) and Subgroup 2 (root non-resorption: root length decrease < 1 mm). Besides, orthodontic patients were further divided into Subgroup A (adolescent, age < 18 years) and Subgroup B (adult, age ≥ 18 years) based on their pre-treatment ages.

### PBF measurements

The PBF in the maxillary and mandibular anterior teeth (canines, lateral incisors, and central incisors) were measured using LDF (PeriFlux 5000, Perimed AB, Stockholm, Sweden), according to the manufacturer’s instructions. The laser wavelength was 780 nm, and the probe fiber separation was 0.5 mm. For orthodontic patients, the PBF was measured at three time points: 1 day after fixed appliance removal (T1), 1 month after fixed appliance removal (T2), and 3 months after fixed appliance removal (T3). For non-orthodontic patients, the PBF was measured to obtain control values.

**Fig. 2:**
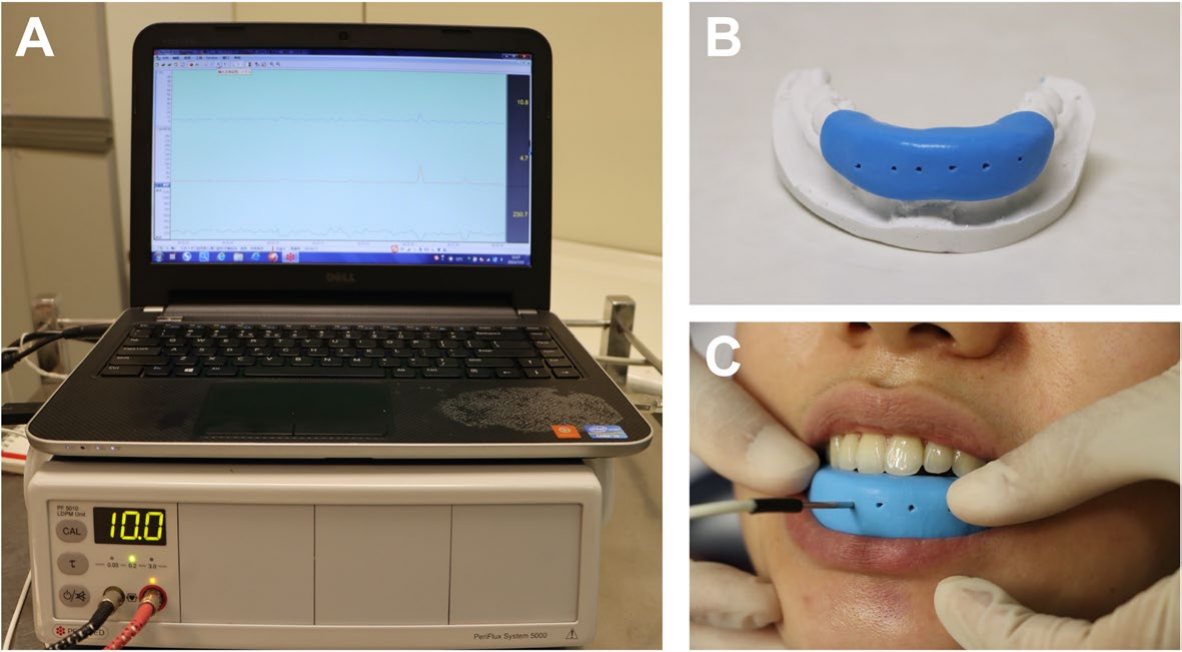
Diagram of pulpal blood flow (PBF) measurement. **A**, laser Doppler flowmetry; **B**, an individual-customized silicone splint; **C**, clinical photograph of PBF measurement

To ensure the accuracy and reproducibility of the LDF probe position on the same tooth surfaces at each time point, an individual customized silicone splint was used in our study. As shown in Fig. 2, a plaster model of each patient was used to construct a two-component silicone splint (Silagum; DMG GmbH, Hamburg, Germany). To avoid signal contamination from gingival tissues, the customized silicone splint with 4–5 mm thickness covered all anterior teeth and the surrounding areas. The hole in the splint was placed 3 mm from the gingival margin and at the mesiodistal center of each anterior tooth. Before PBF measurement, the patient rested in the dental chair for at least 10 min. The probe was inserted vertically into the hole of the silicone splint to contact the tooth surface. The measurement was continued until at least 2 min stable data were recorded. All measurements were performed by the same examiner (QY) under the same environmental conditions. To ensure the accuracy of measurement, the LDF was calibrated once a month using the commercial motility standard (PF1001, Perimed, Stockholm, Sweden), which was a colloidal suspension of latex particles. The LDF data were transferred to Perisoft software (Perimed PSW2.5, Gastrosoft Inc; Jarfalla, Sweden) for further analysis. The mean perfusion units of stable values were calculated as the main outcome. To increase the sample size, the left and right sides of the anterior teeth in each including patient were analyzed separately.

### Statistical analysis

The data were analyzed using SPSS software (version 26; SPSS, Chicago, IL, USA). The Shapiro–Wilk test was used to evaluate the normality of measurement distributions. For normally distributed measurements, repeated-measures analysis of variance with the post-hoc least significant difference test was used to evaluate differences among the three time points. The independent-samples *t* test was used to compare mean differences between groups. The Wilcoxon signed-rank test was performed when the measurement distributions were not normal. Statistical significance was set at *P* < 0.05.

## Results

The mean PBF values for anterior teeth in OG at the three time points and in NG are shown in Table 1. In OG, the PBF in all maxillary anterior teeth exhibited a transient increase at T2 and a decrease at T3; the increase differed significantly only in the maxillary central incisors (*P* = 0.019), in which PBF was significantly higher at T2 (9.24 ± 3.31) than at T1 (7.50 ± 3.17). No significant difference in PBF in the mandibular teeth among the three time points was observed. Compared with those in NG, PBFs in the maxillary lateral incisors, maxillary central incisors, and mandibular lateral incisors at T2 were significantly higher in OG (*P* = 0.048, *P* = 0.04, and *P* = 0.021). No significant difference in PBF was observed between groups at T3, indicating that the PBF in the anterior teeth in patients undergoing extraction had returned to the non-orthodontic level at 3 months after appliance removal.

**Table 1:** The comparison of PBF value in orthodontic patients at three time points and non-orthodontic patients

Teeth	Orthodontic group (OG, *n* = 50)				Non-orthodontic group (NG, ***n*** = 50)	OG T1 vs. NG	OG T2 vs. NG	OG T3 vs. NG
	**T1**	**T2**	**T3**	***P*** ^**a**^ **value**				
**Maxillary Canines**	9.21 ± 4.35	10.74 ± 5.34	10.42 ± 4.36	0.259	9.32 ± 5.06	0.885	0.163	0.102
**Maxillary lateral incisors**	8.87 ± 3.73	9.90 ± 3.49	9.07 ± 2.97	0.338	8.87 ± 4.59	0.579	0.048^*^	0.215
**Maxillary central incisors**	7.50 ± 3.17	9.24 ± 3.31^&^	8.20 ± 3.16	0.019^*^	7.62 ± 3.70	0.896	0.004^*^	0.179
**Mandibular Canines**	11.54 ± 4.59	11.33 ± 4.80	11.08 ± 4.05	0.872	11.04 ± 5.95	0.226	0.416	0.469
**Mandibular lateral incisors**	11.44 ± 4.68	11.95 ± 4.30	11.21 ± 4.72	0.608	9.75 ± 5.01	0.072	0.021^*^	0.121
**Mandibular centrals**	11.70 ± 5.53	11.37 ± 5.12	11.86 ± 4.89	0.855	10.18 ± 4.69	0.234	0.285	0.076

^a^Results of One-way analysis of variance; ^*^ *P* < 0.05; ^&^ T1 vs. T2, *P* < 0.05

The anterior teeth in OG were further divided into Subgroup 1 (root resorption subgroup) and Subgroup 2 (root non-resorption subgroup); the results of PBF comparison between Subgroup 1 and Subgroup 2 are shown in Table 2. The root resorption prevalence was 10% in the maxillary canines, 34% in the maxillary lateral incisors, 50% in the maxillary central incisors, 18% in the mandibular canines, 46% in the mandibular lateral incisors, and 32% in the mandibular central incisors. No significant difference in PBF was found between Subgroup 1 and Subgroup 2 at any of the three time points. In addition, the PBF change during the first 3 months after appliance removal showed no significant difference within subgroups.

**Table 2:** The comparison of PBF value in orthodontic patients between Subgroup 1 (root resorption) and Subgroup 2 (root non-resorption)

Timepoint	Maxillary Canines			Maxillary lateral incisors			Maxillary central incisors			Mandibular Canines			Mandibular lateral incisors			Mandibular central incisors		
	**Subgroup 1** **(** ***n*** **= 5)**	**Subgroup 2** **(** ***n*** **= 45)**	***P*** **value**	**Subgroup 1** **(** ***n*** **= 17)**	**Subgroup 2** **(** ***n*** **= 33)**	***P*** **value**	**Subgroup 1** **(** ***n*** **= 25)**	**Subgroup 2** **(** ***n*** **= 25)**	***P*** **value**	**Subgroup 1** **(** ***n*** **= 9)**	**Subgroup 2** **(** ***n*** **= 41)**	***P*** **value**	**Subgroup 1** **(** ***n*** **= 18)**	**Subgroup 2** **(** ***n*** **= 32)**	***P*** **value**	**Subgroup 1** **(** ***n*** **= 16)**	**Subgroup 2** **(** ***n*** **= 34)**	***P*** **value**
**T1**	9.00 ± 4.08	9.23 ± 4.42	0.207	9.29 ± 4.33	8.66 ± 3.44	0.578	7.13 ± 3.27	7.87 ± 3.09	0.420	11.47 ± 3.83	11.56 ± 4.78	0.957	12.55 ± 3.98	10.82 ± 4.98	0.086	12.04 ± 5.46	11.53 ± 5.63	0.764
**T2**	12.50 ± 4.19	10.54 ± 5.46	0.076	9.74 ± 3.68	9.98 ± 3.44	0.815	8.42 ± 2.72	10.05 ± 3.69	0.081	10.71 ± 5.92	11.46 ± 4.60	0.261	12.64 ± 4.40	11.57 ± 4.26	0.402	11.42 ± 6.14	11.35 ± 4.67	0.811
**T3**	12.05 ± 3.32	10.24 ± 4.45	0.225	9.53 ± 2.82	8.83 ± 3.06	0.440	7.38 ± 2.88	9.02 ± 3.27	0.101	11.18 ± 3.79	11.05 ± 4.15	0.960	11.69 ± 6.45	10.95 ± 3.50	0.600	12.16 ± 5.20	11.72 ± 4.81	0.803
***P*** ^**a**^ **value**	0.613	0.427		0.662	0.216		0.173	0.135		0.717	0.978		0.550	0.758		0.560	0.928	

^a^Results of One-way analysis of variance; ^*^ *P* < 0.05

The orthodontic patients were further divided based on age into Subgroup A (9 adolescent patients; mean age, 14.44 ± 1.33 years) and Subgroup B (16 adult patients; mean age, 26.38 ± 5.12 years). Table 3 shows the comparison of PBF in the anterior teeth between Subgroup A and Subgroup B at the three time points. The PBFs in the maxillary canines, maxillary lateral incisors, mandibular canines, mandibular lateral incisors, and mandibular central incisors at T2 were significantly higher in Subgroup A than Subgroup B (*P* = 0.002, *P* = 0.001, *P* = 0.002, *P* = 0.009, and *P* = 0.011), whereas the PBFs in all anterior teeth at T1 and T3 did not differ significantly between subgroups. In Subgroup A, the maxillary lateral incisors had a higher PBF at T2 (12.23 ± 3.48) than at T1 (9.10 ± 3.76) and T3 (9.81 ± 2.80) with statistically significant difference (*P* = 0.020). In Subgroup B, the PBF did not differ significantly during the first 3 months after appliance removal.

**Table 3:** The comparison of PBF value in orthodontic patients between Subgroup A (adolescent) and Subgroup B (adult)

Timepoint	Maxillary Canines			Maxillary lateral incisors			Maxillary central incisors			Mandibular Canines			Mandibular lateral incisors			Mandibular central incisors		
	**Subgroup A** **(** ***n*** **= 18)**	**Subgroup B** **(** ***n*** **= 32)**	***P*** **value**	**Subgroup A** **(** ***n*** **= 18)**	**Subgroup B** **(** ***n*** **= 32)**	***P*** **value**	**Subgroup A** **(** ***n*** **= 18)**	**Subgroup B** **(** ***n*** **= 32)**	***P*** **value**	**Subgroup A** **(** ***n*** **= 18)**	**Subgroup B** **(** ***n*** **= 32)**	***P*** **value**	**Subgroup A** **(** ***n*** **= 18)**	**Subgroup B** **(** ***n*** **= 32)**	***P*** **value**	**Subgroup A** **(** ***n*** **= 18)**	**Subgroup B** **(** ***n*** **= 32)**	***P*** **value**
**T1**	10.24 ± 4.94	9.75 ± 7.18	0.505	9.10 ± 3.76	8.74 ± 3.77	0.747	7.50 ± 3.56	7.50 ± 2.99	0.777	11.07 ± 5.53	11.81 ± 4.04	0.587	11.44 ± 5.59	11.44 ± 4.18	0.995	12.34 ± 7.11	11.34 ± 4.49	0.595
**T2**	14.06 ± 5.51	8.87 ± 4.29	0.002^*^	12.23 ± 3.48 ^&^	8.70 ± 2.87	0.001^*^	10.14 ± 4.09	8.73 ± 2.73	0.289	14.02 ± 5.62	9.81 ± 3.54	0.002^*^	14.31 ± 4.89	10.63 ± 3.33	0.009^*^	14.17 ± 6.08	9.80 ± 3.75	0.011^*^
**T3**	11.70 ± 3.30	9.70 ± 4.75	0.120	9.81 ± 2.80	8.65 ± 3.03	0.191	8.51 ± 2.38	8.03 ± 3.55	0.612	11.55 ± 4.08	10.81 ± 4.07	0.544	12.49 ± 5.58	10.50 ± 4.09	0.436	12.97 ± 5.67	11.24 ± 4.37	0.233
***P*** ^a^ **value**	0.055	0.777		0.020^*^	0.994		0.075	0.289		0.189	0.127		0.277	0.574		0.677	0.269	

^a^ Results of One-way analysis of variance; ^*^ *P* < 0.05

## Discussion

The response of dental pulp to orthodontic force has been discussed widely. A series of changes in dental pulp could occur after force application, such as circulatory disturbance, edema, vacuolization, and fibrohyalinosis [4, 5]. Javed et al. and Weissheimer T et al. systematically reviewed the influence of orthodontic treatment on dental pulp, and both concluded that the evidence is insufficient to prove that orthodontic treatment causes irreversible changes to the dental pulp [27, 28]. Hence, the state of dental pulp should be monitored during and after orthodontic treatment. Among the pulp vitality tests, the PBF measurement could provide an accurate and reliable assessment of pulp status [14]. LDF is a well-documented and noninvasive method of directly evaluating PBF without causing pulp damage and relying on patient’s response [13].

Recent studies have used LDF to evaluate the PBF change during the early phase of orthodontic treatment [11, 19, 20, 22, 29–31]. Brodin et al. and Sabuncuoglu et al. both demonstrated that intrusive force could temporarily decrease the PBF of anterior teeth [19, 20]. Ersahan et al. reported that the decrease of PBF was also reversible during the intrusion of the maxillary first molar [11]. Elham S. et al. found that there was no significant difference in PBF changes between conventional and self-ligating fixed appliances, which both reduced within 48 h, increased after one week and returned to original level after one month [22]. Another study also conducted by Elham S. et al. revealed that the PBF changes caused by clear aligners and fixed appliances were not significantly different [31]. These studies all indicated that the PBF tends to decrease transiently before returning to the baseline level several weeks later. After orthodontic force application, a temporary disruption in the pulp blood supply may occur due to the compression of periapical vessels, which is associated with a decrease in oxygen tension; this type of disruption increases the risk of cellular injury in pulp tissue [15, 16]. However, the PBF observation durations in these studies were relatively short; thus, their results reflect only the short-term effect of orthodontic treatment on PBF. Prolonged force application and the long-distance movement of anterior teeth may jeopardize pulp vitality. In the current study, we analyzed the PBF in the maxillary and mandibular anterior teeth after orthodontic retraction.

In our study, the PBF in the maxillary and mandibular anterior teeth after orthodontic retraction was similar to that in non-orthodontic patients, confirming that orthodontic retraction did not jeopardize the dental pulp blood supply. Although the PBF has been reported to decrease temporarily after orthodontic force application, it eventually returns to the non-orthodontic level. Interestingly, we also observed a temporary increase in the PBF in the maxillary anterior teeth 1 month after appliance removal, probably due to the alleviation of vascular compression caused by tooth movement. Blood vessel dilation occurs temporarily after the removal of orthodontic force; however, the apical circulation recovers gradually with the regeneration of pulp vessels. The PBF in the maxillary anterior teeth returned to the non-orthodontic level at 3 months after appliance removal. The transient increase in PBF after appliance removal was more significant in the maxillary anterior teeth than in the mandibular anterior teeth. One possible explanation for this difference is that the former underwent more apical movement. Apical movement of the mandibular incisors is limited due to the thinness of the surrounding alveolar bone. Hence, the PBF in the mandibular anterior teeth was relatively stable during the first 3 months after appliance removal.

Dental pulp responses may vary according to the type of tooth movement and the magnitude of orthodontic force [30, 32]. Previous studies have focused mainly on the effect of intrusive force on PBF [18, 20, 29]. Ikawa et al. and Sano et al. reported that intrusive force caused a temporary reduction in PBF in the maxillary incisors, with a return to the baseline level several weeks later [18, 29]. At present, only one study has involved analysis of the response of dental pulp to retraction force [21]. Sabuncuoglu et al. reported that the PBF in the maxillary canines increased immediately after retraction force application, decreased 1 week later, and returned to a near-baseline level 1 month later. Hence, the changes in the dental pulp blood supply during intrusive and retraction movements may differ. The magnitude of dental pulp inflammation is commonly related to that of orthodontic force. Greater orthodontic force is recognized to cause more significant PBF reductions. Sabuncuoglu et al. reported that the PBF was reduced significantly after intrusion with great force (120 g) compared with the use of a slighter force (40 g) [20]. A finite element study also indicated that the stress at apical third of pulp during the teeth movement is smaller than that at neurovascular bundle, a light force did not compromise the PBF [33]. Hence, the use of slight force to induce tooth movement is necessary to minimize the damage to the dental pulp and decrease the occurrence of root resorption. In this study, the retraction force was 150 g, applied at 1-month intervals.

The change in dental pulp status during orthodontic treatment is complex, with a series of biological events in pulp tissue triggered by orthodontic force. The response of pulp tissue to orthodontic force has been reported to exhibit a transition from acute to chronic inflammatory conditions [27, 28]. The pulp blood vessels experience three phases after orthodontic force application: hyperemia, ischemia, and recovery. The release of neurotransmitters, cytokines, and metabolites, such as epithelial growth factor and interleukin 1, play an important role in pulp blood supply changes, especially the angiogenic response [34–36]. Force application is re-activated monthly over the course of orthodontic treatment, leading to cycles of inflammation. Under these conditions, the dental pulp tissue, like other connective tissues, has an elevated repair capacity and can adapt to the orthodontic force [37]. The dental pulp has been reported to show an extraordinary ability to withstand a long-term continuous heavy force [32]. On the other hand, orthodontic tooth movement could stimulate odontoblasts to form secondary dentin. The 3-D volumetric assessment of dental pulp space could be achieved by several medical imaging tools [38]. Several studies have shown that the pulp cavity volume in the anterior teeth decreases after orthodontic treatment [7, 39]. The incidence of pulp stone formation was also found to increase, as orthodontic force promotes pulp tissue calcification [40]. These changes in tissue mineralization may further affect the dental pulp blood supply. Taken together, our results indicate that the adaptation of dental pulp tissue during orthodontic treatment eventually results in a steady blood flow.

Root resorption caused by orthodontic force is common, especially in patients who have undergone four premolars extraction. The amount of root displacement is an essential factor in root resorption [41]. Consequently, there is a high incidence of root resorption in anterior teeth with significant retraction. As the root resorption is a change in three-dimensional morphology, the CBCT was used in our study due to its superior accuracy and sensitivity in detecting root resorption [42]. We found that the prevalence of root resorption in the maxillary and mandibular anterior teeth ranged from 10 to 50%, consistent with previous findings [43, 44]. In this study, we first analyzed the PBF in anterior teeth with and without root resorption. Although the apical foramina of the anterior teeth were open in the root resorption subgroup, PBF after appliance removal did not differ significantly between subgroups. A possible explanation for this finding is that the restoration of the pulp blood supply occurred at the end of the orthodontic treatment. The PBF was relatively stable regardless of whether the apical foramen was open. It is worth mentioning that no severe root resorption (loss of more than one-third of the root length) was observed in this study. The maximum amount of root resorption in our patients was 2.67 mm. On the other hand, root resorption presumably stops after the removal of orthodontic force [45]. The open apical foramen could subsequently narrow with the physiological formation of secondary dentin. Hence, mild to moderate root resorption caused by orthodontic treatment may not influence the dental pulp status. Notably, our results are inconsistent with those of Younessian et al., who reported a correlation between root resorption and electric pulp test (EPT) responses during orthodontic treatment and found a significant association between decreases in EPT responses and increased root resorption [46]. Methodological differences contributed to the inconsistency of results between studies. Younessian et al. measured root resorption by periapical radiography, whereas we diagnosed it by CBCT. Compared with the EPT, PBF measurement is more suitable for the assessment of dental pulp health.

Age-related changes in dental pulp circulation also play an important role in the dental pulp status during orthodontic treatment. A histological study has shown that the PBF decreases with age due to reductions in the number of blood vessels and amount of pulp [47]. With increasing age, the formation of secondary dentin and cementum could lead to the narrowing of the pulp chamber and root apex, which would compromise dental blood flow and the lymphatic and nervous systems. Hence, the dental pulp blood supply in adolescent and adult patients may differ. Additionally, the thickness of mineralized tissue may influence light penetration into teeth during LDF measurements. At present, only one study has investigated the effect of age on dental pulp circulation during 1 month of orthodontic treatment; it revealed that the decrease in PBF was more severe in older patients than in younger patients [23]. In our study, we analyzed the effect of age on dental pulp circulation after orthodontic treatment. After the removal of orthodontic force application, transient blood vessel dilation was more significant in adolescent than in adult patients. This difference is probably attributable to the high blood supply to pulp tissue in adolescent patients. Although the PBF in the anterior teeth during the 3-month period after orthodontic treatment was higher in adolescent patients than in adult patients, the difference was not significant.

### Limitations

This study is not without limitations. First, the sample was relatively small. Two sides of the dentition were analyzed separately to double the sample size. Second, the pre-treatment PBF values for the orthodontic group was lacked, which prohibited determination of whether the PBF returned to the baseline level after orthodontic extraction treatment. For this reason, non-orthodontic patients served as the control group. The PBF at T3 did not differ significantly between orthodontic and non-orthodontic patients. Our results indicated that the en mass retraction of maxillary and mandibular anterior teeth with an appropriate force level would not compromise the dental pulp health. Future studies with large sample size and pre- and post-treatment PBF values are required to confirm our results. Third, although the pulp blood supply determined by LDF is the most accurate indicator of dental pulp health, the threshold value of blood supply triggering pulp necrosis remains unclear.

## Conclusion

The PBF in the maxillary and mandibular anterior teeth at 1 day after orthodontic appliance removal was not significantly different from that in non-orthodontic patients.

The PBF in the maxillary central incisors had increased transiently at 1 month after orthodontic appliance removal and returned to the non-orthodontic level 3 months later.

The PBF in the maxillary and mandibular anterior teeth after orthodontic retraction may not be influenced by root resorption.

The transient increase in PBF at 1 month after appliance removal was more obvious in adolescent than in adult patients.

## Data Availability

All data analyzed during the current study are included in this published article. Any other request about the data, contact e-mail: weiranli@bjmu.edu.cn.

## References

[1] Huang YP, Li WR (2015). Correlation between objective and subjective evaluation of profile in bimaxillary protrusion patients after orthodontic treatment. Angle Orthod.

[2] Chan EK, Soh J, Petocz P, Darendeliler MA (2008). Esthetic evaluation of Asian-Chinese profiles from a white perspective. Am J Orthod Dentofacial Orthop.

[3] Nixon CE, Saviano JA, King GJ, Keeling SD (1993). Histomorphometric study of dental pulp during orthodontic tooth movement. J Endod.

[4] Vitali FC, Cardoso IV, Mello FW, Flores-Mir C, Andrada AC, Dutra-Horstmann KL (2021). Effect of orthodontic force on dental pulp histomorphology and tissue factor expression. Angle Orthod.

[5] Lazzaretti DN, Bortoluzzi GS, Torres Fernandes LF, Rodriguez R, Grehs RA, Martins Hartmann MS (2014). Histologic evaluation of human pulp tissue after orthodontic intrusion. J Endod.

[6] Mostafa YA, Iskander KG, El-Mangoury NH (1991). Iatrogenic pulpal reactions to orthodontic extrusion. Am J Orthod Dentofacial Orthop.

[7] Popp TW, Artun J, Linge L (1992). Pulpal response to orthodontic tooth movement in adolescents: a radiographic study. Am J Orthod Dentofacial Orthop.

[8] Hamersky PA, Weimer AD, Taintor JF (1980). The effect of orthodontic force application on the pulpal tissue respiration rate in the human premolar. Am J Orthod.

[9] Vitali FC, Cardoso IV, Mello FW, Flores-Mir C, Andrada AC, Dutra-Horstmann KL (2022). Association between Orthodontic Force and Dental Pulp Changes: A Systematic Review of Clinical and Radiographic Outcomes. J Endod.

[10] Veberiene R, Smailiene D, Danielyte J, Toleikis A, Dagys A, Machiulskiene V (2009). Effects of intrusive force on selected determinants of pulp vitality. Angle Orthod.

[11] Ersahan S, Sabuncuoglu FA (2015). Effects of magnitude of intrusive force on pulpal blood flow in maxillary molars. Am J Orthod Dentofacial Orthop.

[12] Abd-Elmeguid A, Yu DC (2009). Dental pulp neurophysiology: part 2. Current diagnostic tests to assess pulp vitality. J Can Dent Assoc.

[13] Fratkin RD, Kenny DJ, Johnston DH (1999). Evaluation of a laser Doppler flowmeter to assess blood flow in human primary incisor teeth. Pediatr Dent.

[14] Karayilmaz H, Kirzioğlu Z (2011). Comparison of the reliability of laser Doppler flowmetry, pulse oximetry and electric pulp tester in assessing the pulp vitality of human teeth. J Oral Rehabil.

[15] Perinetti G, Varvara G, Festa F, Esposito P (2004). Aspartate aminotransferase activity in pulp of orthodontically treated teeth. Am J Orthod Dentofacial Orthop.

[16] Perinetti G, Varvara G, Salini L, Tetè S (2005). Alkaline phosphatase activity in dental pulp of orthodontically treated teeth. Am J Orthod Dentofacial Orthop.

[17] Salles AW, Salles AM, Nogueira GE (2013). Laser Doppler Blood-Flow Signals from Human Teeth during an Alignment and Leveling Movement Using a Superelastic Archwire. ISRN Dent.

[18] Ikawa M, Fujiwara M, Horiuchi H, Shimauchi H (2001). The effect of short-term tooth intrusion on human pulpal blood flow measured by laser Doppler flowmetry. Arch Oral Biol.

[19] Brodin P, Linge L, Aars H (1996). Instant assessment of pulpal blood flow after orthodontic force application. J Orofac Orthop.

[20] Sabuncuoglu FA, Ersahan S (2014). Changes in maxillary incisor dental pulp blood flow during intrusion by mini-implants. Acta Odontol Scand.

[21] Sabuncuoglu FA, Ersahan S (2016). Changes in human pulp blood flow during canine retraction. Acta Odontol Scand.

[22] Abu Alhaija ES, Taha NA (2021). A comparative study of initial changes in pulpal blood flow between conventional and self-ligating fixed orthodontic brackets during leveling and alignment stage. Clin Oral Investig.

[23] Ersahan S, Sabuncuoglu FA (2018). Effect of age on pulpal blood flow in human teeth during orthodontic movement. J Oral Sci.

[24] Zhang X, Zhou H, Liao X, Liu Y (2022). The influence of bracket torque on external apical root resorption in bimaxillary protrusion patients: a retrospective study. BMC Oral Health.

[25] Li J, Zhao Y, Li H, Li H, Lei L (2018). Effects of force magnitude on torque control in the correction of bimaxillary protrusion with mass retraction. J Orthod Sci.

[26] Lu YZX, Gao X (2009). Comparison of the friction resistance in sliding mechanics at different forces level for space closing—a nonlinear finite element study. Chin J Orthod.

[27] Weissheimer T, Silva E, Pinto KP, So GB, Rosa RA, So MVR (2021). Do orthodontic tooth movements induce pulp necrosis? A systematic review. Int Endod J.

[28] Javed F, Al-Kheraif AA, Romanos EB, Romanos GE (2015). Influence of orthodontic forces on human dental pulp: a systematic review. Arch Oral Biol.

[29] Sano Y, Ikawa M, Sugawara J, Horiuchi H, Mitani H (2002). The effect of continuous intrusive force on human pulpal blood flow. Eur J Orthod.

[30] Sübay RK, Kaya H, Tarim B, Sübay A, Cox CF (2001). Response of human pulpal tissue to orthodontic extrusive applications. J Endod.

[31] Abu Alhaija ESJ, Al-Abdallah SY, Taha NA (2019). A comparative study of initial changes in pulpal blood flow between clear aligners and fixed orthodontic appliances. Am J Orthod Dentofacial Orthop.

[32] Han G, Hu M, Zhang Y, Jiang H (2013). Pulp vitality and histologic changes in human dental pulp after the application of moderate and severe intrusive orthodontic forces. Am J Orthod Dentofacial Orthop.

[33] Moga RA, Cosgarea R, Buru SM, Chiorean CG (2019). Finite element analysis of the dental pulp under orthodontic forces. Am J Orthod Dentofacial Orthop.

[34] Derringer K, Linden R (2007). Epidermal growth factor released in human dental pulp following orthodontic force. Eur J Orthod.

[35] Derringer KA, Jaggers DC, Linden RW (1996). Angiogenesis in human dental pulp following orthodontic tooth movement. J Dent Res.

[36] Derringer KA, Linden RW (2004). Vascular endothelial growth factor, fibroblast growth factor 2, platelet derived growth factor and transforming growth factor beta released in human dental pulp following orthodontic force. Arch Oral Biol.

[37] Cuoghi OA, Faria LP, Ervolino E, Barioni SRP, Topolski F, Arana-Chavez VE (2018). Pulp analysis of teeth submitted to different types of forces: a histological study in rats. J Appl Oral Sci.

[38] Shetty H, Shetty S, Kakade A, Shetty A, Karobari MI, Pawar AM (2021). Three-dimensional semi-automated volumetric assessment of the pulp space of teeth following regenerative dental procedures. Sci Rep.

[39] Venkatesh S, Ajmera S, Ganeshkar SV (2014). Volumetric pulp changes after orthodontic treatment determined by cone-beam computed tomography. J Endod.

[40] Ertas ET, Veli I, Akin M, Ertas H, Atici MY (2017). Dental pulp stone formation during orthodontic treatment: A retrospective clinical follow-up study. Niger J Clin Pract.

[41] Heboyan A, Avetisyan A, Karobari MI, Marya A, Khurshid Z, Rokaya D (2022). Tooth root resorption: A review. Sci Prog.

[42] Lee YJ, Kook YA, Park JH, Park J, Bayome M, Vaid NR (2019). Short-term cone-beam computed tomography evaluation of maxillary third molar changes after total arch distalization in adolescents. Am J Orthod Dentofacial Orthop.

[43] Dudic A, Giannopoulou C, Leuzinger M, Kiliaridis S (2009). Detection of apical root resorption after orthodontic treatment by using panoramic radiography and cone-beam computed tomography of super-high resolution. Am J Orthod Dentofacial Orthop.

[44] Deng Y, Sun Y, Xu T (2018). Evaluation of root resorption after comprehensive orthodontic treatment using cone beam computed tomography (CBCT): a meta-analysis. BMC Oral Health.

[45] Abbott PV, Lin S (2022). Tooth resorption-Part 2: A clinical classification. Dent Traumatol.

[46] Younessian F, Behnaz M, Badiee M, Dalaie K, Sarikhani A, Shekarian S (2021). The correlation between external apical root resorption and electric pulp test responses: a prospective clinical trial. Dent Press J Orthod.

[47] Bernick S (1967). Age changes in the blood supply to human teeth. J Dent Res.

